# Novel robot-assisted minimally invasive surgical technique for the treatment of esophageal cancer

**DOI:** 10.20452/wiitm.2024.17916

**Published:** 2024-11-28

**Authors:** Michał Wiłkojć, Wojciech Migal, Agnieszka Majewska, Anna Różańska-Walędziak

**Affiliations:** Department of General, Oncological, Metabolic and Thoracic Surgery, Military Institute of Medicine – National Research Institute, Warszawa, Poland; Interdisciplinary Student Association of Metabolic and Systemic Diseases “Salus Aegroti,” Medical Faculty, Collegium Medicum, Cardinal Stefan Wyszyński University in Warsaw, Warszawa, Poland; Department of Human Physiology and Patophysiology, Faculty of Medicine, Collegium Medicum, Cardinal Stefan Wyszynski University in Warsaw, Warszawa, Poland

**Keywords:** esophageal cancer, Ivor Lewis, robot-assisted minimally invasive esophagectomy, robotic surgery

## Abstract

A new trend toward the application robot-assisted minimally invasive esophagectomy (RAMIE) has started to develop in the field of gastrointestinal tract surgery. As compared with the current gold standard, minimally invasive esophagectomy, RAMIE facilitates more thorough upper mediastinal lymphadenectomy and reduces the risk of vessel damage. This study aimed to evaluate the technical feasibility and safety of RAMIE in patients with esophageal cancer based on the first 2 RAMIE procedures carried out in Poland. Both robotic procedures were successful, without the need for conversion to open surgery. There was no case of incomplete lesion removal or postoperative complications, indicating that RAMIE is a feasible and safe treatment option for patients with esophageal cancer and results in good postoperative recovery.

## INTRODUCTION 

Surgical techniques used in the treatment of esophageal cancer have evolved over the years. With the development of microinvasive techniques, the previously common open esophagectomy approach has been gradually replaced by laparoscopic esophagectomy. The first evoltionary step in the field of esophageal cancer surgery was the development of minimally invasive esophagectomy (MIE), performed through a laparoscopic transperitoneal approach. The next step was combining 2 laparoscopic phases of the procedure, abdominal and thoracic. The rigid thorax, which is hard to access during the thoracoscopic phase due to its proximity to the ribs, scapula and spine, proved to be a particularly challenging environment for standard minimally invasive instruments.

The positive evidence for the safety and efficacy of MIE in terms of procedure time, complication rate, and length of hospital stay, led to the recent development of robot-assisted minimally invasive esophagectomy (RAMIE), a technique that allowed for overcoming the technical limitations encountered with MIE.^1^ Advanced robotic instruments with a great range of motion, built-in tremor filtration systems, and improved magnification enable surgeons to perform more precise dissection in tighter spaces.

Esophageal cancer is the seventh most common cancer worldwide in terms of morbidity, and sixth in terms of mortality.^2^ In recent years, there has been a change in the prevalence of different histologic subtypes of esophageal cancer, with a decrease in the incidence of squamous cell carcinoma and an increase in the incidence of adenocarcinoma.

## AIM 

The purpose of this study was to present a novel robot-assisted minimally invasive surgical technique for the treatment of esophageal cancer. The procedure was performed in 2 two patients, and this is the first description of its application in Poland.

## MATERIALS AND METHODS 

### Patients 

The study included 2 consecutive patients with histopathologically confirmed esophageal cancer who underwent a RAMIE Ivor Lewis procedure using a laparoscopic abdominal and right thoracoscopic approach between January and February 2024. All robotic procedures were performed by a single surgeon (MW) with long-standing experience in laparoscopic and robotic surgery, and the da Vinci Xi surgical system was used (Intuitive, Sunnyvale, California, United States). Both patients provided informed consent for the procedure and for publishing their case descriptions. We evaluated the course of the surgical procedure and the surgical outcomes, including operative time, need for conversion to a laparoscopic or open approach, length of hospital stay, and incidence of postoperative complications.

### Surgical technique 

The robot-assisted Ivor Lewis esophagectomy is performed via a laparoscopic abdominal and right thoracoscopic approach. The lower and middle parts of the esophagus, up to the level of the azygos vein, are resected en bloc with the subcardial portion of the stomach. Gastroesophageal anastomosis is created using a gastric conduit pull-up formed from the greater curvature of the stomach and displaced into the thoracic cavity, which is followed by the creation of a robot-sewn intrathoracic anastomosis. Indocyanine green fluorescence angiography is used to evaluate local vascularity. In the subsequent phase of the procedure, abdominal lymphadenectomy including the paracardial, left gastric, common hepatic, splenic, and celiac nodes is performed, followed by thoracic lymphadenectomy that includes the upper and lower paratracheal, subcarinal, paraesophageal, and pulmonary ligament lymph nodes. The abdominal stage of the surgery can be performed using an open, 3-dimensional (3D) laparoscopic, or robotic approach. In the case of our patients, the robotic approach was used.

### Abdominal phase (robotic) 

During this phase, the patient is placed in the supine position, with arms along the body and legs split. Three 8-mm ports and a single 12-mm port are put in place under direct vision in a horizontal line above the umbilicus (within about 6 cm distance of each other), and an additional 12-mm trocar for the assistant is inserted in the right mesogastrium. The patient is placed in the reverse Trendelenburg position at a 15 ° angle, and the robotic cart is docked on the right side of the patient. Dissection is performed using a monopolar cautery hook and bipolar forceps, with additional use of a bipolar vessel sealer device.

The first stage of the surgery involves dissection of the greater gastric curvature along the gastrocolic ligament, followed by mobilization of the stomach up to the left crura, while sparing the right gastroepiploic arcade. The short gastric arteries, as well as any existing retrogastric adhesions, are dissected and ligated. Next, a full Kocher maneuver is performed and the gastrohepatic ligament is cut open close to the liver and up to the right crus of the diaphragm, while preserving the right gastric artery. Lymph node dissection is performed along the upper margin of the common hepatic artery and up to the celiac axis. In the next stage, the right crus of the diaphragm is transected to enlarge the esophageal hiatus, and a gastric tube of about 4 cm in diameter is created on the side of the greater curvature with a stapler. Perfusion of the created gastric tube is verified with indocyanine green fluorescence, and the divided stomach is sewn to the end of the divided esophagus, with a marker suture left for the next stage of the surgery. The abdominal phase ends with jejunostomy and placement of a drain.

### Thoracic phase (robotic) 

The thoracic phase starts with the introduction of single-lung ventilation. The patient is placed in the left lateral decubitus position at a 45 ° angle, with the robotic cart still docked on the right side. Four 8-mm robotic ports in the third, fifth, seventh, ninth intercostal space along the edge of the latissimus dorsi muscle and a single 12-mm port in the sixth intercostal space (for the assistants) are placed. A 7–8 mm Hg pneumothorax is induced, and the parietal pleura is cut from the level of the azygos arch down to the diaphragm, starting at the anterior side of the esophagus. The pulmonary ligament is divided at the level of the diaphragm, the azygos arch is sectioned, and right paratracheal lymphadenectomy is performed, followed by deepened dissection of the parietal pleura toward the esophageal hiatus to expose the aorta. The right vagus nerve and its bronchial branches are sectioned. The esophagus is dissected below the tracheal bifurcation, a drain is placed around it, and the dissection is extended to the level of the diaphragm, followed by clipping the thoracic duct. The lower and middle parts of the esophagus, up to the level of the azygos vein, are resected en bloc with the subcardial portion of the stomach and the periesophageal, bronchial, and subcarinal lymph nodes. The proximal remaining part of the esophagus is divided above the level of the azygos vein, and the esophago-gastric bloc with the gastric conduit are pulled through the previously widened esophageal hiatus, until the marker suture is visible. Then, the esophagogastric bloc is disconnected from the gastric conduit and removed by an enlarged assistant port. The esophageal mucosa is everted by placing stitches between the mucosa and external muscular layer of the esophagus. The proximal esophagus is dilated with a Foley catheter, and perfusion in the esophagus and gastric conduit is verified with indocyanine green fluorescence. The proximal part of the gastric conduit is cut at more than 2 cm from the stapler line, and a single-layer esophagogastric anastomosis is performed above the level of the azygos arch with 2 separate running, self-locking barbed sutures, from the posterior to the anterior surface. The anastomosis is checked for leaks with methylene blue, and a chest drain is inserted via a robotic trocar port, with the apex placed in the upper chest, posteriorly to the anastomosis. The remaining ports are

then closed with stitches.

## RESULTS 

The first patient was a 54-year-old man diagnosed with adenocarcinoma in the lower third part of the esophagus in December 2023. Initially, esophageal submucosal dissection was performed without an adequate surgical margin, as the tumor was found to have infiltrated the submucosa layer. The patient was admitted to the Department of General, Oncological, Metabolic, and Thoracic Surgery of the Military Medical Institute in Warsaw for complementary surgical excision of esophageal cancer. He was qualified for a RAMIE Ivor Lewis procedure, which was successfully performed on January 30, 2024. Postoperative histopathologic examination revealed no lymph node metastases. Follow-up gastroscopy performed on the tenth day after the surgery revealed a widediameter unrestricted anastomosis without any signs of fistula, and oral alimentation was introduced. The patient was discharged home in good condition after 18 days of hospitalization without any adverse events.

The second patient was a 70-year-old man with chronic renal disease and a history of appendectomy and cholecystectomy. Due to gastrointestinal bleeding, he underwent endoscopy, during which a tumor in the lower third part of the esophagus was found. After histopathologic examination, the tumor was diagnosed as esophageal adenocarcinoma. The patient was admitted to the surgical department for radical esophageal resection, and was qualified and prepared for RAMIE. The Ivor Lewis esophagectomy was performed without complications on February 14, 2024. Postoperative histopathologic examination revealed metastasis in one of the resected lymph nodes. Following the surgery, the patient was stable but required renal dialysis therapy due to chronic renal disease, which resulted in anuria in the postoperative period. Contrast radiography performed 9 days after the surgery revealed a wide-diameter unrestricted anastomosis, and oral alimentation was introduced. The patient was discharged after 15 days of hospitalization. On follow-up examination performed on April 3, 2024, he was in good condition, with normal complete blood count results. Gastroscopy showed no changes or abnormalities in the anastomosis. The patient was scheduled for adjuvant chemoradiation therapy according to the protocol for esophageal cancer with lymph node metastases.

## DISCUSSION 

In March 2024, a meta-analysis of 18187 patients with a history of surgical treatment for esophageal cancer was conducted to determine the current contribution of RAMIE to the surgical management of this patient population.^3^ The purpose of the study was to compare perioperative complications and short-term surgical outcomes associated with RAMIE and MIE. Surgical outcomes included the operative time, estimated blood loss, conversion rate to open surgery, lymph node retrieval, and 30- and 90-day mortality rates. Postoperative outcomes comprised anastomotic and chyle leakage, recurrent laryngeal nerve palsy, pulmonary, cardiac, and infectious complications, and length of hospital stay. RAMIE was found to be a safe and feasible alternative to conventional MIE, with a tendency toward superiority in blood loss, lymph node yield, pulmonary complications, and length of hospital stay. Of note, there was significant heterogeneity among the included studies for some of the outcomes measured.

Other studies^4^^;^^5^ aimed to establish which surgical approach (RAMIE, MIE, or open surgery) was the safest and associated with the lowest risk of complications. In terms of procedure duration, RAMIE is more time-consuming than MIE. The learning curve for RAMIE is about 20 procedures before the operative time is reduced.^6^^;^^7^

The estimated blood loss is lower after RAMIE than after MIE, and both techniques are associated with reduced blood loss, as compared with the open approach.^4^^;^^5^^;^^8^^;^^9^ The superiority of RAMIE in terms of blood loss may be explained by improved instrument dexterity in navigating the surgical field and its tremor-filtering systems.

The RAMIE technique allows for performing a more meticulous lymphadenectomy than MIE or open esophagectomy, with a greater number of lymph nodes that can be harvested.^3^^;^^10^ This is attributable to the lower risk of vessel damage associated with RAMIE, as compared with MIE or open esophagectomy. In a study by Deng et al,^11^ which compared lymphadenectomy during RAMIE and MIE, the RAMIE group exhibited a greater number of overall resected lymph nodes. The greatest difference in the number of resected lymph nodes was observed in the upper mediastinum, particularly along the left recurrent laryngeal nerve.^11^ Therefore, in the case of RAMIE, the learning curve affects not only the operative time but also the lymph node yield.^6^ Approximately 20 procedures are required to be performed in order to reach the level of surgical proficiency in RAMIE. Also, significantly higher numbers of upper mediastinal lymph nodes as well as left and right recurrent laryngeal nerve lymph nodes were observed to have been additionally retrieved in subsequent procedures, and the lymph node yield was observed to increase up to the 60th procedure performed.^7^ Lymph node metastases represent the most frequent site of secondary tumor development in esophageal cancer following radical treatment. Thus, RAMIE, which permits the excision of additional mediastinal and abdominal lymph nodes, may be regarded as a favorable prognostic indicator for patients with esophageal cancer.

The conversion rate to open surgery as well as 30- and 90-day mortality rates are comparable between MIE and RAMIE.3,10 There are no significant differences with respect to long-term survival and 3-year overall survival between MIE and RAMIE; however, RAMIE is associated with a higher 3-year disease-free survival rate.^9^^;^^10^ For both techniques, the 1- and 5-year survival rates are higher than those reported for open surgery.^5^

As compared with classic open surgery, apart from being associated with reduced blood loss and higher numbers of lymph nodes resected, RAMIE and MIE are related to fewer pulmonary complications, and the rate of such complications seems to be lower after RAMIE than after MIE.^5^^;^^8^ The rate of pulmonary complications is an important factor contributing to long-term survival.^12^

In terms of anastomosis leaks, another complication associated with decreased long-term survival, as well as chyle leakage, recurrent laryngeal nerve palsy, cardiac complications, and infectious complications, no significant differences were noted between RAMIE, MIE, and open surgery.^3^^;^^13^

The duration of hospitalization, an outcome important both for the patient and the health care system, is the shortest after RAMIE, as compared with MIE and open surgery.^1^^;^ ^14^The level of pain experienced by patients following surgery plays a crucial role in shaping their overall quality of life, as it directly impacts their physical comfort, emotional well-being, and ability to engage in daily activities. Previous studies showed that patients treated with RAMIE reported significantly less pain, both in the 3-month^15^ and 2-year follow-up, as compared with those who underwent open thoracic esophagectomy. This emphasizes the benefits of robot-assisted minimally invasive surgery in improving postoperative pain management and enhancing patient recovery and satisfaction.^16^ There are no data enabling a comparison of the pain level after surgery between MIE and RAMIE, which highlights the necessity for a comprehensive study on the subject to provide information about the efficacy of both techniques in minimizing postoperative pain and improving patient quality of life.

To date, no study has directly analyzed the cost-effectiveness of RAMIE in relation to other surgical techniques. Some studies indicate that the higher cost for health care systems incurred by robot-assisted procedures can be balanced by the lower postoperative complication rates and shorter hospital stays, but without further details.^14^^;^^17^ Exploring the economic implications, including the cost of hospitalization and postoperative care as well as the potential for complications, could provide valuable insights into the financial feasibility and long-term sustainability of incorporating RAMIE into clinical practice.

There are still other aspects of minimally invasive treatment of esophageal cancer, such as the influence of body weight. Wang et al^18^ pointed out that high body mass index was associated with prolonged operative time and increased blood loss in MIE. However, it was not related to the rate of postoperative complications, and was not identified as an independent prognostic factor for survival in esophageal squamous cell carcinoma patients undergoing MIE. It is worth mentioning that lymphovascular invasion was associated with shorter disease-specific survival and was found to be an independent prognostic factor after MIE in node-negative patients with esophageal squamous cell carcinoma.^19^

## CONCLUSIONS 

RAMIE with robot-sewn intrathoracic anastomosis appears to be a feasible, safe, and effective treatment method, with favorable perioperative results. In the patients with esophageal cancer eligible for the procedure, the RAMIE technique seems to be the optimal treatment option, with the lowest complication rate and the highest success rate in radical lymphad-enectomy. The successful outcomes of the first surgeries in Poland, as well as the current data showing that RAMIE is a safe and beneficial alternative to MIE, give light to the possibility of establishing this method as a mainstay of treatment for esophageal cancer also in Poland. However, this would require implementation of specialized educational programs for surgeons to reduce the learning curve as well as increasing the accessibility of robotic systems.
